# FUNCTIONING OUTCOMES AT OLDER WORKING AGES BY INCOME GROUPS: COMPARING THE US WITH 11 EUROPEAN COUNTRIES

**DOI:** 10.1093/geroni/igae098.0442

**Published:** 2024-12-31

**Authors:** HwaJung Choi, Stephen Jivraj, Stefan Fors

**Affiliations:** University of Michigan, Ann Arbor, Michigan, United States; University College London, London, England, United Kingdom; Karolinska institutet, Stockholm, Stockholms Lan, Sweden

## Abstract

This study extends prior research by examining physical and cognitive function for low vs. high-income working-age adults (55-64 years) in the U.S. by comparing to 11 European countries. Using a pooled cross-sectional analysis, the study estimated differences in comparably measured outcomes of activities of daily living (ADLs), instrumental activities of daily living (IADLs), and word recall assessment, with adjustment for survey design aspects and sociodemographic characteristics. Income groups were determined based on the distribution within each country, year, and age. Overall, older working-age adults from the U.S. were significantly more likely to have difficulty with ADLs and IADLs compared to all European countries examined in the study, with the exception of having a similar outcome with England for having difficulty with ADLs. In particular, the adjusted prevalence of ADL limitation from the lowest income group (bottom 20%) was 30% in the U.S. vs. 4%-13% in 10 European countries. Those from the highest income group (top 20%) in the U.S. had a similar risk of having difficulty with ADLs to most countries examined in the study but had a greater risk of having difficulty with IADLs compared to the majority of the countries in the study. Word recall scores from the lowest income group were poorer in the U.S. compared to many European countries, while those from the highest income group performed better in the U.S. Targeting the poorest in the U.S. at risk of disability through intervention should be a priority.

